# Supplementary material for: Genome-Wide Association Study on Imputed Genotypes of 180 Eurasian Soybean Glycine max Varieties for Oil and Protein Contents in Seeds
Source: Plants (Basel). 2025 Jan 17;14(2):255. doi: 10.3390/plants14020255 (PMC11768550; doi:10.3390/plants14020255)
Supplement: Supplementary file 1 [file plants-14-00255-s001.zip › Suppl_Figures.pdf]

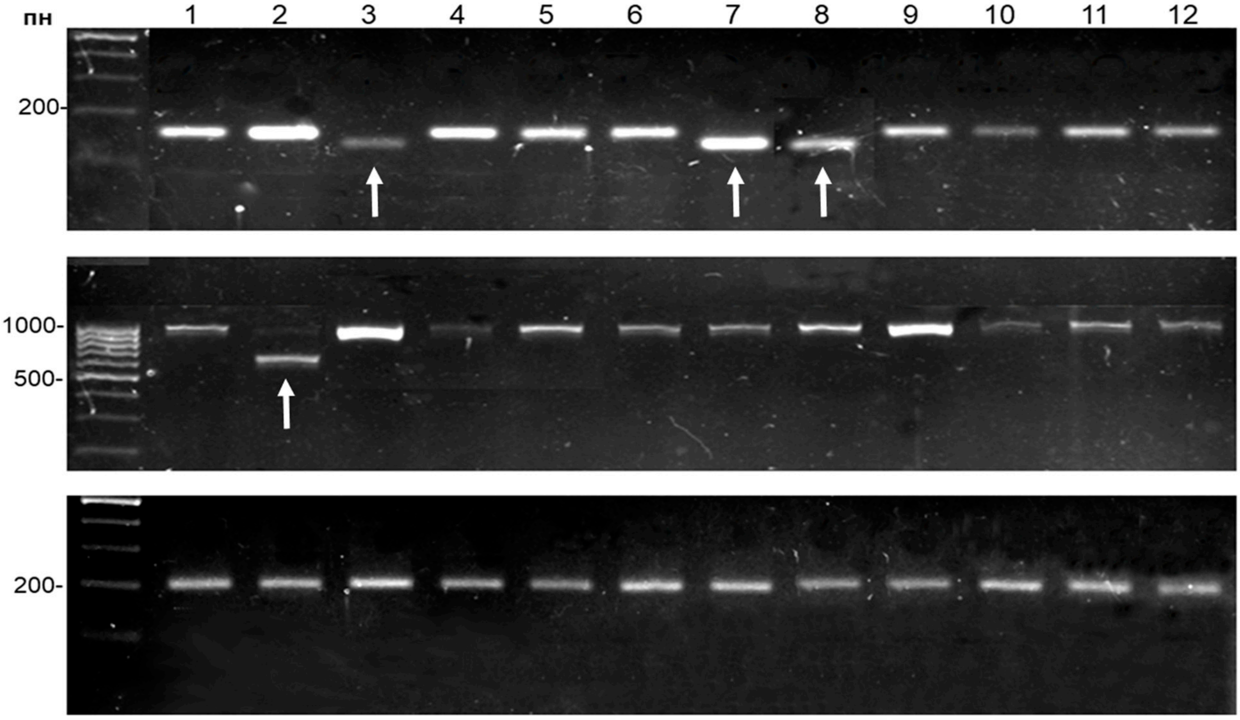

Supplementary Figure S1. PCR results of Russian soybean cultivars using markers for the genes: *GmST05, POWR1,* and *GmSWEET39* (from top to bottom).1 – Gorinskaya; 2 – KA 14/18 (*Glycine soja*);
3 – Gribskaya 12; 4 – SibNIIK-9; 5 – SNK-285; 6 – Kruzhevnitsa; 7 – Svetlaya;
8 – Belgorodskaya 48; 9 – SNK-315; 10 – Persona; 11 – Yankan; 12 – Aktai. Arrows indicate the products of the "high-protein" alleles of the genes.

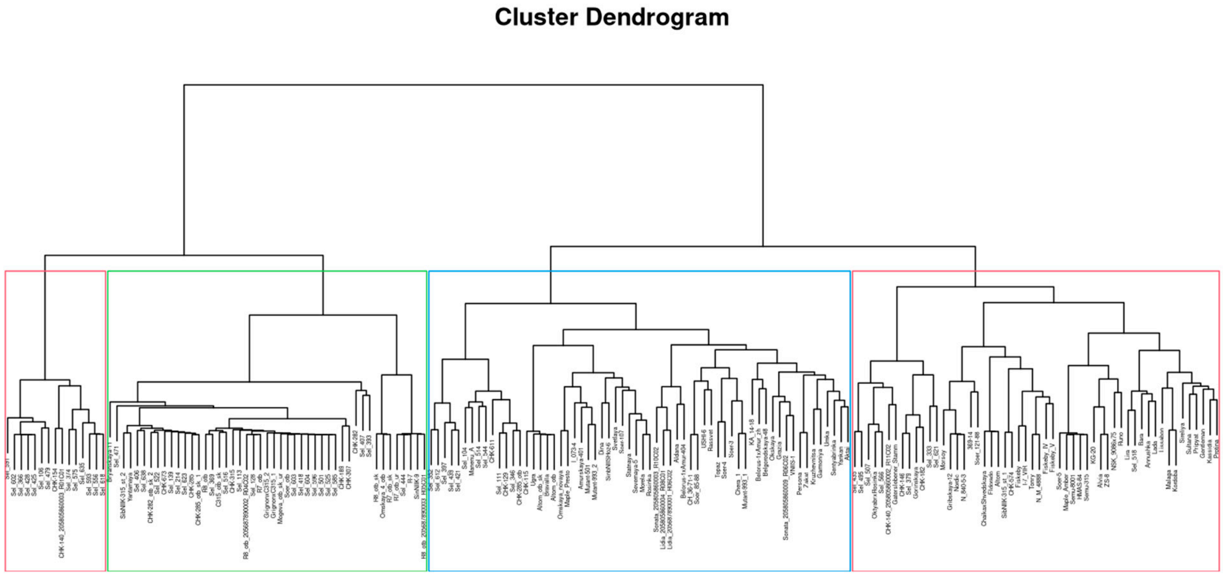

Supplementary Figure S2. Cluster dendrogram constructed on genomic relationships between studied lines and varieties reflects similar pattern of clusterization as it was shown in [7].

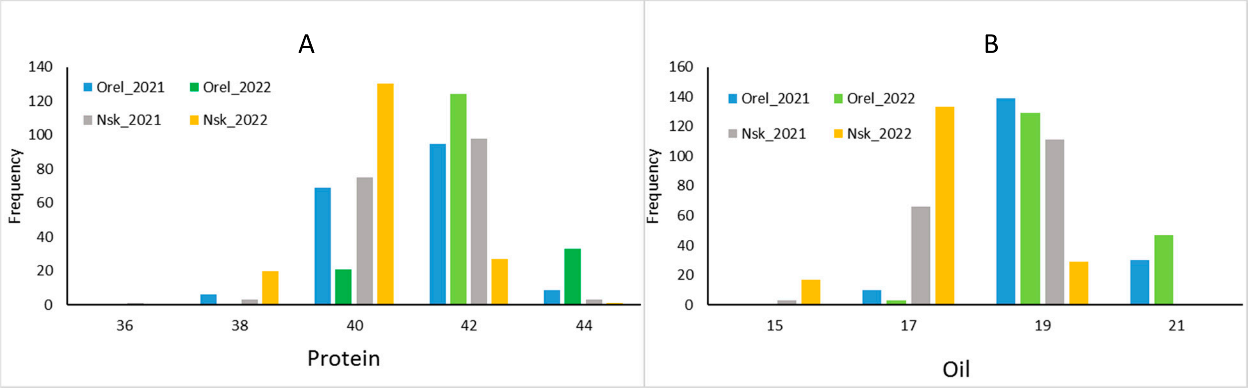

Supplementary Figure S3. Distribution of protein and oil content in Novosibirsk (Nsk) (A) and Orel (B) regions in 2021 and 2022.

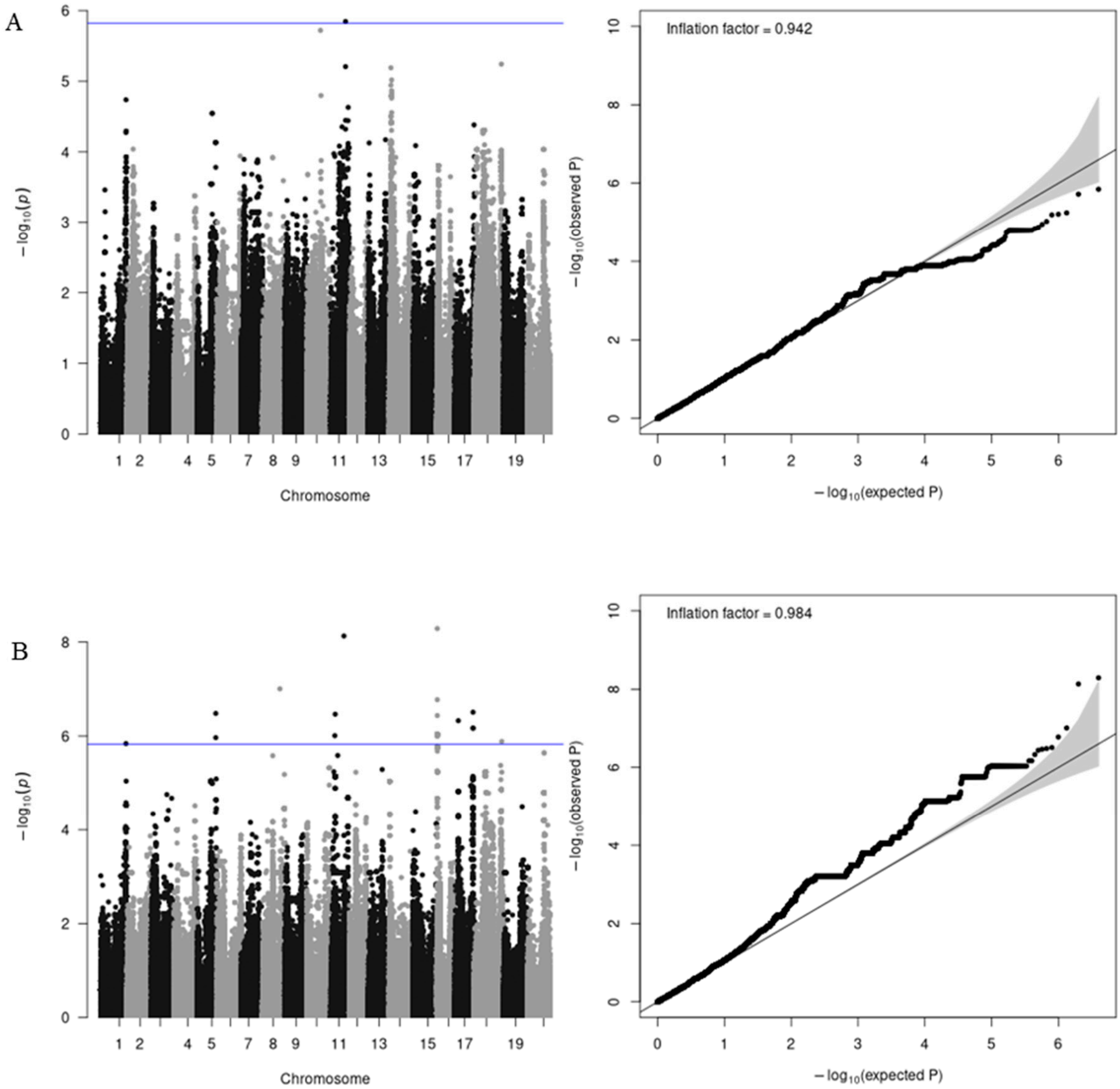

Supplementary Figure S4. Manhattan and QQ plots for protein content GWAS results in Novosibirsk (A) and Orel (B) regions. The plots represent the −log10 transformed p-values. The horizontal blue line indicates the significance level (1.5×10^{-6}).

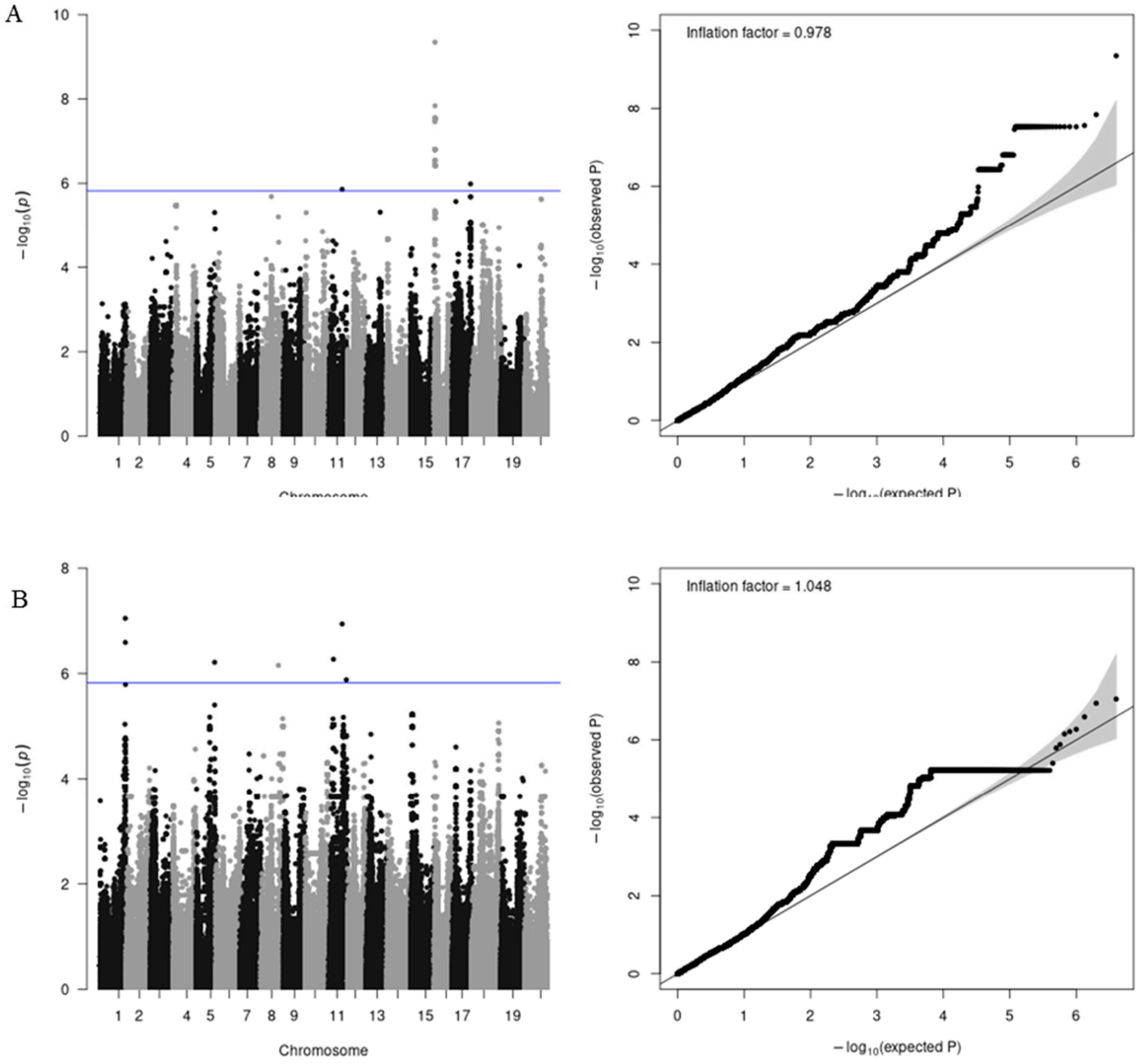

Supplementary Figure S5. Manhattan and QQ plots for oil content GWAS results in Novosibirsk (top) and Orel (bottom) region. The plots represent the −log10 transformed p-values. The horizontal blue line indicates the significance level (1.5×10^{-6}).
